# FPD2GB2: Automating a transition from a customized genome browser to GBrowse2

**DOI:** 10.1186/1471-2105-14-S17-A17

**Published:** 2013-10-22

**Authors:** Roger Chui, Jerzy W Jaromczyk, Neil Moore, Christopher L Schardl

**Affiliations:** 1 Department of Computer Science, University of Kentucky, Lexington, KY 40506, USA; 2 Department of Plant Pathology, University of Kentucky, Lexington, KY 40546, USA

## Background

We present FPD2GB2, a collection of scripts to automate data migration of a custom genome database and browser to a new implementation. The fungal endophytes genome project (http://www.endophyte.uky.edu/) hosted at the University of Kentucky uses a custom genome browser and database to support several genome sequencing, annotation, and analysis projects. This system is currently based around a locally-developed genome annotation database, visualized using the GBrowse version 1 genome browser together with a large amount of custom code that supports metadata and rendering features not supported by GBrowse 1. Since our initial development of our genome site, a newer version of GBrowse, version 2, has been released. Unlike GBrowse version 1, version 2 supports a very expressive database schema based on the standardized GFF3 format. This schema natively supports the types of data that we currently store in our custom database. In order to simplify maintenance, to ease the upgrade path to future versions of GBrowse, and to improve interoperability with software and websites that support GBrowse and the GFF3 format, we have decided to migrate our custom database to the GFF3 format supported by GBrowse 2. This migration allows us to simplify and in some cases even eliminate much of our custom code. We discuss the challenges presented by such a data migration task, and describe the FPD2GB2 script collection we developed to automate the process.

